# Supplementary figures and images for: Local retinoic acid signaling directs emergence of the extraocular muscle functional unit
Source: PLoS Biol. 2020 Nov 17;18(11):e3000902. doi: 10.1371/journal.pbio.3000902 (PMC7707851; doi:10.1371/journal.pbio.3000902)

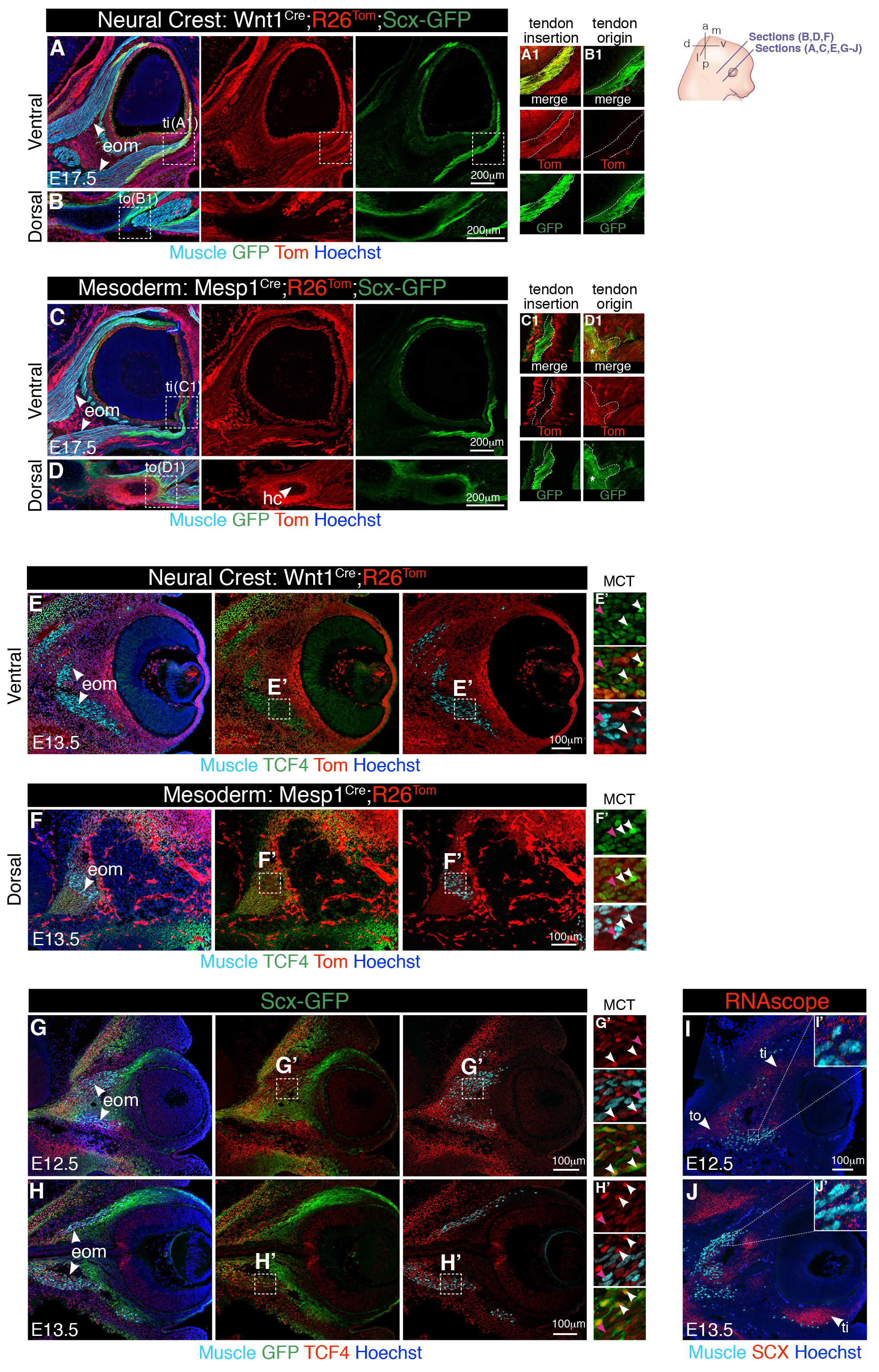

Supplement: S1 Fig — (A-D) NCC (Tg:Wnt1Cre;R26Tom;Scx-GFP) and mesoderm (Mesp1Cre;R26Tom;Scx-GFP) lineage contributions to the periocular region on coronal cryosections of E17.5 embryos, combined with immunostaining for tendon (GFP) and muscle (Tnnt3, Troponin T Type 3, differentiated muscle). Sections at ventral (A,C) and dorsal (B,D) levels. Note that tendon insertions at the level of the orbit are NCC-derived (A), whereas the tendon origin (D) is mesoderm-derived. Higher-magnification views at the level of the tendon insertion (A1,C1) and origin (B1,D1) are shown as insets. Asterisk in D1 indicates Scx-GFP+ cells in the perichondrium of the hypochiasmatic cartilage. (E-F’) NCC (Tg:Wnt1Cre;R26Tom) and mesoderm (Mesp1Cre;R26Tom) lineage contributions to the periocular region on coronal cryosections of E13.5 embryos, combined with immunostaining for TCF4 and muscle (PAX7/MYOD/MYOG, myogenic markers). Note that TCF4 is expressed robustly in connective tissue fibroblasts and at lower levels in myogenic cells. White arrowheads in (E’,F’) mark Tom+, TCF4+, myogenic marker-negative connective tissue cells in the NCC- and mesoderm-derived areas. Pink arrowheads in (E’,F’) mark myogenic cells (TCF4low). (G-H’) Coronal cryosections of E13.5 Tg:Scx-GFP embryos, combined with immunostaining for TCF4 (connective tissue, myogenic progenitors) and muscle (PAX7/MYOD/MYOG). White arrowheads in (G’,H’) mark Tom+, TCF4+, myogenic marker-negative connective tissue cells in the NCC and mesoderm-derived areas. Pink arrowheads in (G’,H’) mark myogenic cells (TCF4low). (I,J) In situ hybridization on E12.5 (I) and E13.5 (J) coronal cryosections for Scx combined with immunofluorescence for muscle (PAX7/MYOD/MYOG). High levels of Scx mRNA are seen at the tendon origin and insertion but also in the bulk of the muscle masses (I’,J’, insets). a, anterior; d, dorsal; l, lateral; m, medial; MCT, muscle connective tissue; E, embryonic day; eom, extraocular muscle; NCC, neural crest cell; p, posterior; ti, tendon inse [file pbio.3000902.s001.tif]

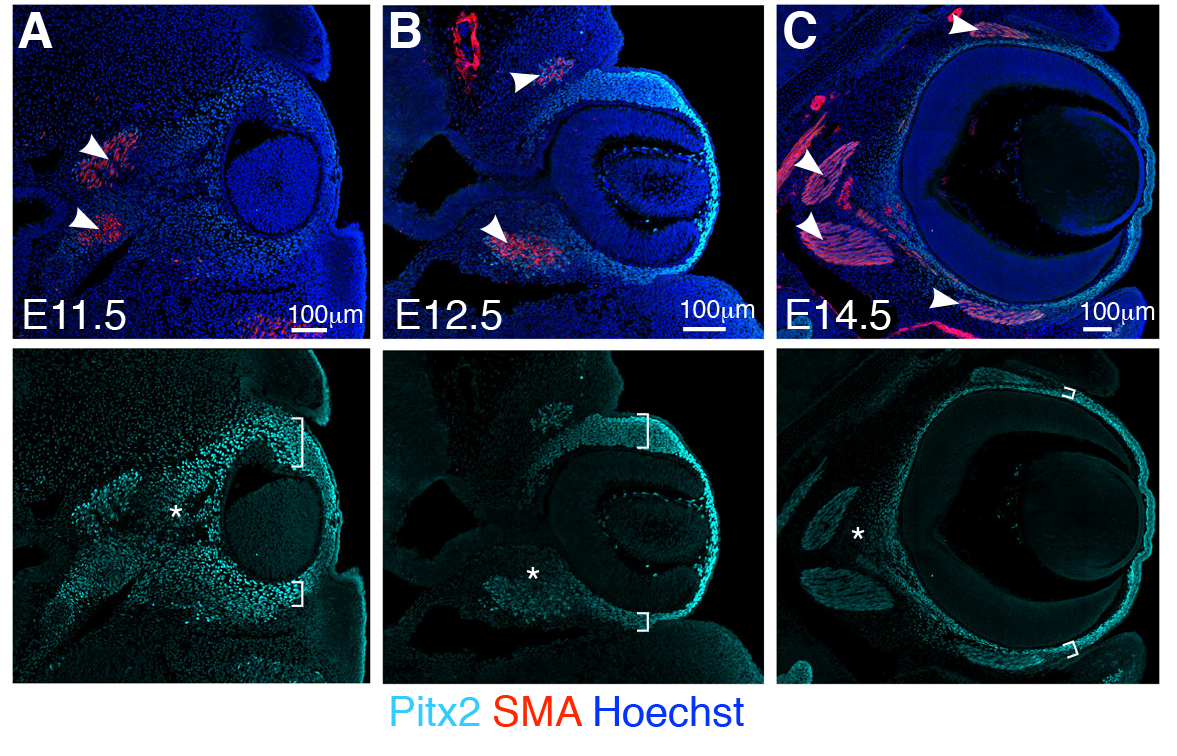

Supplement: S2 Fig — (A-C) Immunostaining on E11.5 (A), E12.5 (B) and E13.5 (C) coronal sections of control embryos for PITX2 (EOM myogenic progenitors, POM) and SMA (differentiated muscle). Arrowheads mark the EOM masses and asteriks point to PITX2 expression in the medial POM. Note thinning of the lateral POM as development proceeds (brackets). (n = 3 per stage). E, embryonic day; EOM, extraocular muscle; POM, periocular mesenchyme. (TIF) [file pbio.3000902.s002.tif]

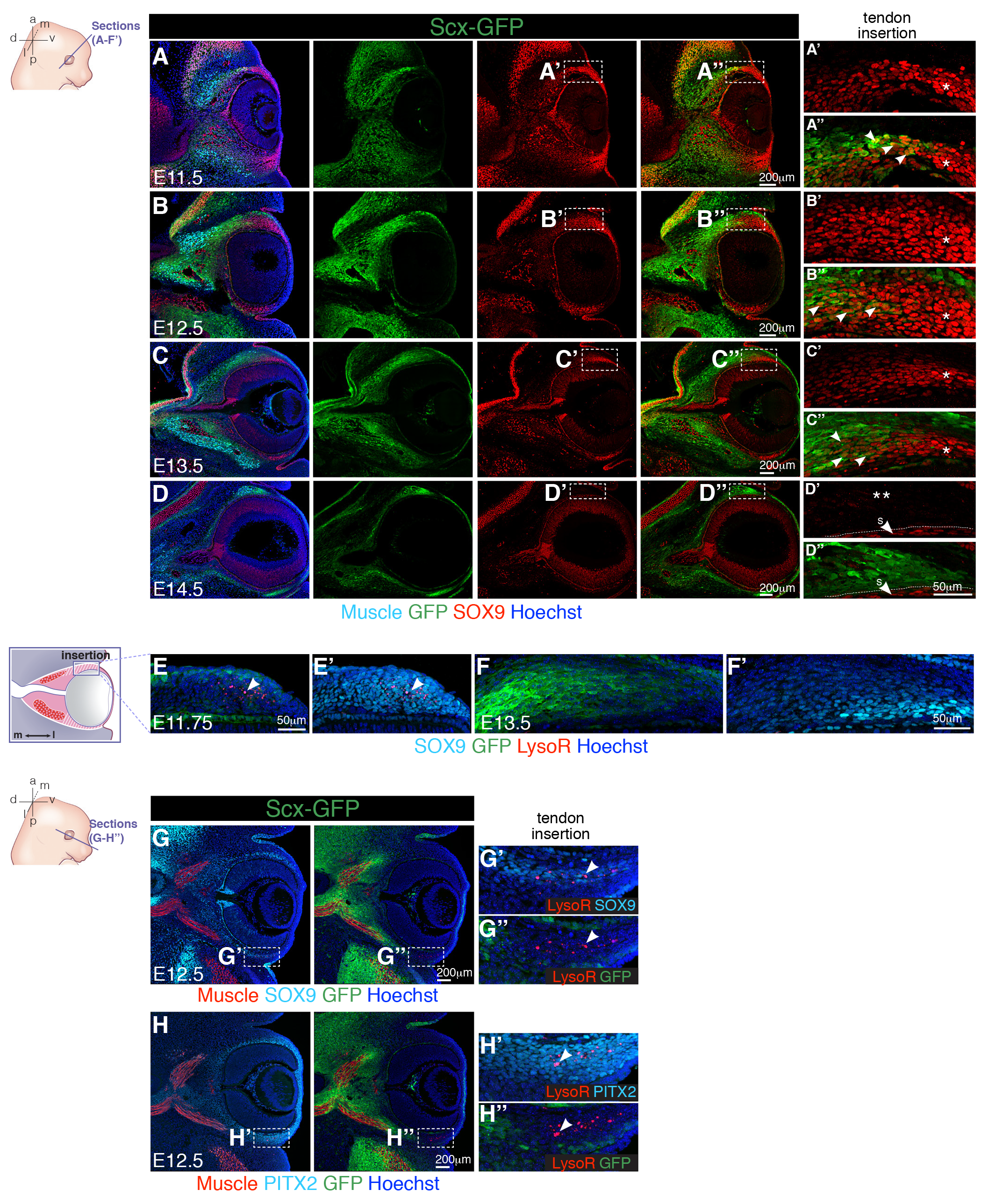

Supplement: S3 Fig — (A-D’) Immunostaining for the indicated markers on coronal sections of E11.5 (A), E12.5 (B, E, F), E13.5 (C) and E14.5 (D) Tg:Scx-GFP embryos. (A’-D”) Higher-magnification views of the anterior tendon insertions (superior rectus) in the POM. White arrowheads mark SOX9+ Scx-GFP+ cells up to E13.5. Asteriks in A’-C” point to SOX9+ Scx-GFP-negative areas. Double asterisk in D’ mark Scx-GFP+ SOX9-negative tendon tips at E14.5. SOX9 expression remains at the sclera. (E-F’) Immunostaining for the indicated markers on coronal sections of E11.75 and E13.5 Tg:Scx-GFP embryos preincubated with LysoR. Arrowheads in E,E’ indicate SOX9+ LysoR+ cells in the E11.75 POM. (G-H”) Immunostaining for the indicated markers on tranversal sections of E12.5 Tg:Scx-GFP embryos. (G’-H”) Higher magnification views of the lateral tendon insertions (lateral rectus) showing SOX9+ or PITX2+ LysoR+ areas. MYOD/MYOG (myogenic markers) in A-D and MyHC (myofibers) in G-H were used to identify the EOMs. (n = 3 per stage). a, anterior; d, dorsal; E, embryonic day; EOM, extraocular muscle; l, lateral; LysoR, LysoTracker Red; m, medial; p, posterior; POM, periocular mesenchyme; s, sclera; v, ventral. (TIF) [file pbio.3000902.s003.tif]

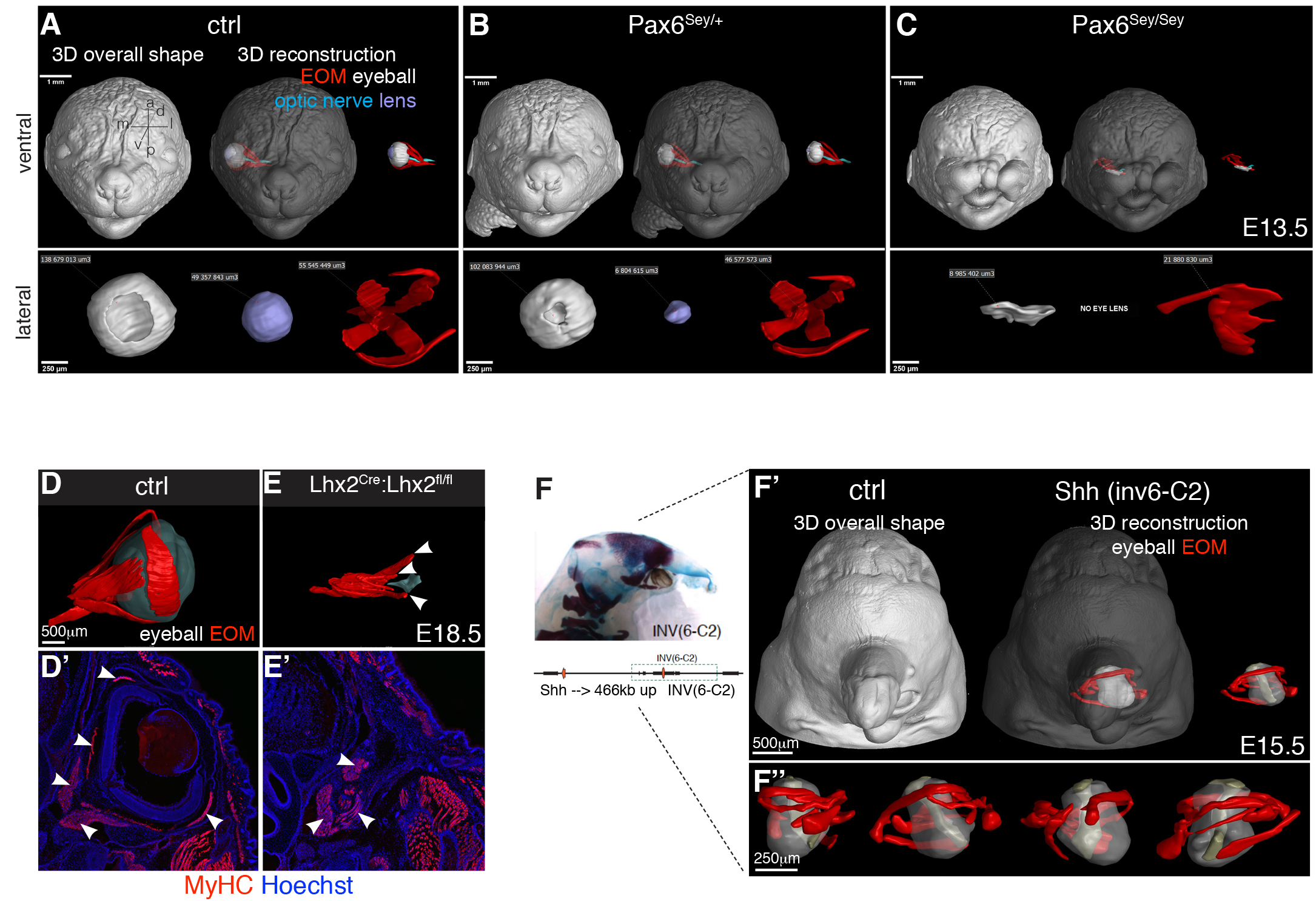

Supplement: S4 Fig — (A-C) Micro-CT-based 3D reconstruction of EOM, eyeball, optic nerve, and lens in E13.5 control (A), Pax6Sey/+ (B), and Pax6Sey/Sey embryos (C). Note that in heterozygote embryos, EOM patterning proceeds normally despite having a smaller retina and lens (n = 3). (D-E) Micro-CT-based 3D-reconstruction of EOM and eyeball in E18.5 control (A) and Tg:Lhx2Cre;Lhx2fl/fl (B) embryos. Arrowheads highlight some extent of EOM segregation in the mutant. (D’-E’) Coronal sections of control (D’) and mutant (E’) embryos stained with MyHC (differentiated muscle). Arrowheads indicate individual EOM masses (n = 2). (F-F”) Analysis of EOM patterning in E15.5 embryos containing inversions of Shh genomic regulatory regions (Inv(6-C2)). (F) Skeletal preparation of mutant embryos displaying cyclopia. (F’-F”) Micro-CT-based 3D-reconstruction of EOM and eyeball of mutant embryos (n = 2). a, anterior; ctrl, control; d, dorsal; E, embryonic day; EOM, extraocular muscle; l, lateral; m, medial; micro-CT, micro-computed tomography; p, posterior; v, ventral. (TIF) [file pbio.3000902.s004.tif]

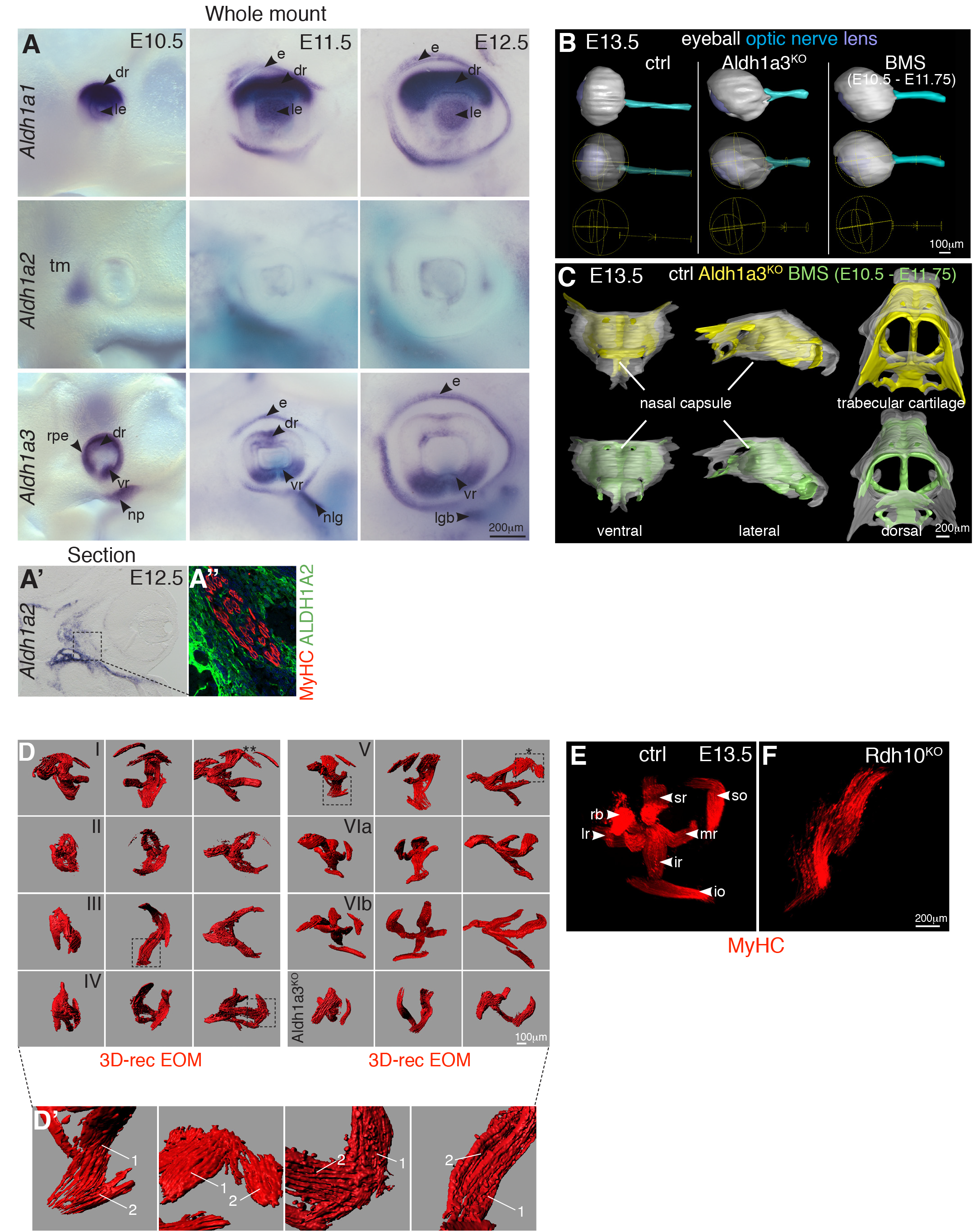

Supplement: S5 Fig — (A) Whole-mount in situ hybridization for Aldh1a1, Aldh1a2, and Aldh1a3 in E10.5, E11.5, and E12.5 wildtype embryos (n = 3). (A’-A”) In situ hybridization for Aldh1a2 (A’) and immunostaining for ALDH1A2 and MyHC (differentiated muscle) (A”) on E12.5 coronal sections. ALDH1A2 is expressed in the temporal mesenchyme and adjacent connective tissues (n = 3). (B) Micro-CT-based 3D-reconstruction of eyeball, optic nerve and lens of E13.5 control, Aldh1a3KO and BMS493-treated embryos (n = 2 each genotype). The lower row is a scheme of a sphere fitting the eyeball and lens, and a cylinder for the optic nerve. Note ventralization of the eyeball in Aldh1a3KO and BMS493-treated embryos. (C) Micro-CT-based 3D-reconstruction of the mesenchymal condensations of the nasal capsule and trabecular cartilage of E13.5 control (white), Aldh1a3KO (yellow) and BMS493-treated (green) embryos. (D-D’) MyHC WMIF of E13.5 BMS493-treated embryos as described in S1 Table. The most severe phenotype obtained in each condition is shown as an isosurface. Two different examples of the phenotype observed upon treatment VI (a,b) are shown (most and least severe). An Aldh1a3KO embryo is shown as comparison. Asterisk denotes ectopic duplicated SO muscle. (D’) Higher-magnification views from D (dashed squares) with examples of adjacent, nonsplit muscle masses (1,2) depicted by differential fiber orientation. (E-F) MyHC WMIF of E13.5 control (E) and Rdh10KO (F) embryos. Note total absence of muscle splitting in mutant (n = 3 per condition). EOMs in D-F were segmented from adjacent head structures and 3D-reconstructed in Imaris (Bitplane). ctrl, control; dr, dorsal retina; 3D-rec, 3D-reconstruction; e, eyelid groove; E, embryonic day; EOM, extraocular muscle; io, inferior oblique; ir, inferior rectus; le, lens; lgb, lacrimal gland bud; lr, lateral rectus; micro-CT, micro-computed tomography; mr, medial rectus; nlg, nasolacrimal groove; np, nasal pit; os, optic stalk; rb, retractor bulbi; rpe, retinal pigmen [file pbio.3000902.s005.tif]

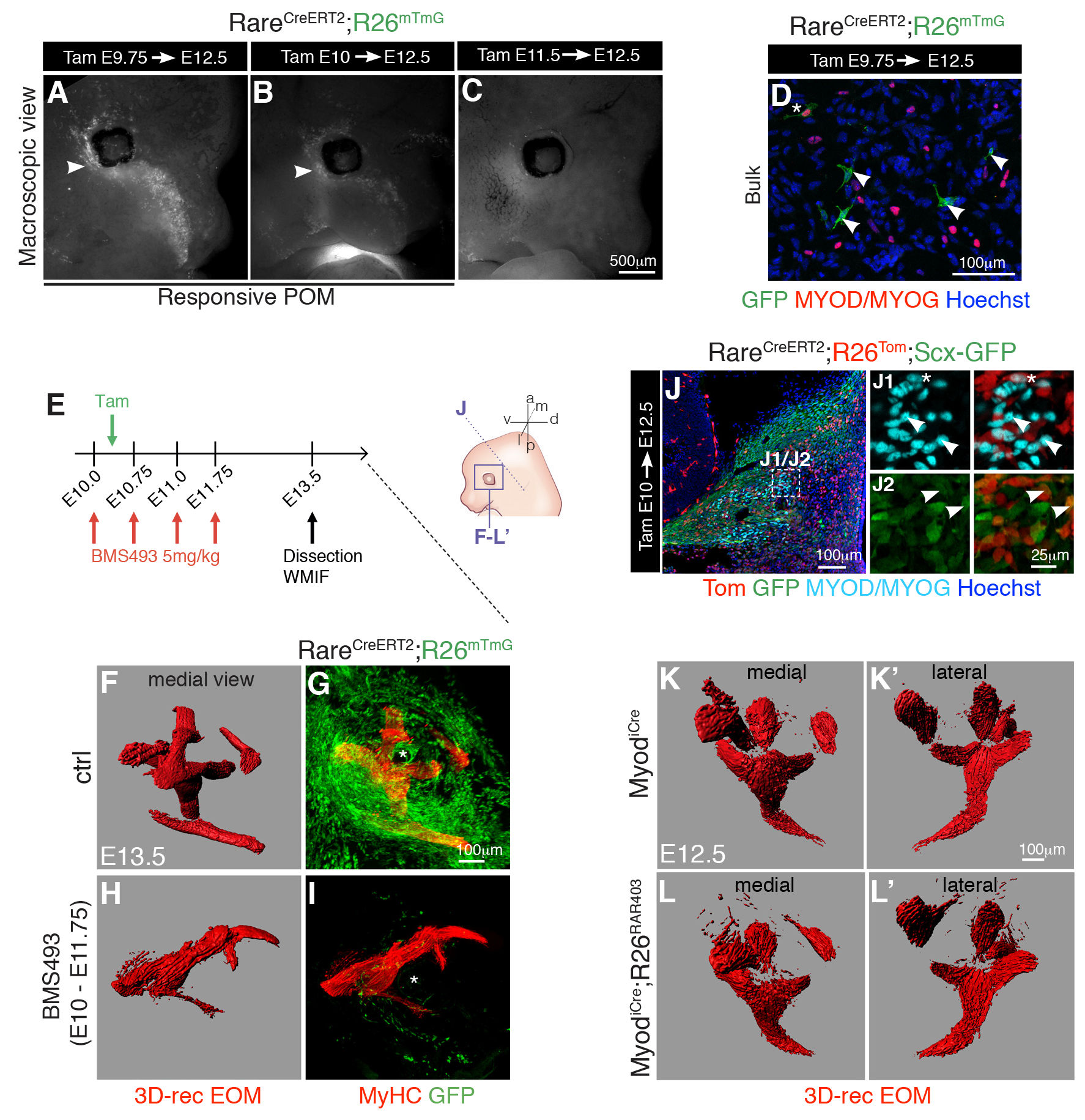

Supplement: S6 Fig — (A-C) Macroscopic views of endogenous GFP fluorescence of Tg:RARE-CreERT2;R26mTmG embryos. Tam was injected into pregnant females and embryos analyzed at indicated time points. Arroheads indicate labeling in POM (n > 3 per condition). (D) Immunostaining on cells isolated from the periocular region of Tg:RARE-CreERT2;R26mTmG embryos for GFP and myogenic markers (MYOD/MYOG). (E) Strategy used to determine responsiveness of Tg:RARE-CreERT2 reporter in presence of BMS493. BMS493 was injected to pregnant females every 10–12 hours between E10 and E11.75. Recombination was induced by tamoxifen at E10.5 (2 hours after the first BMS injection). (F-I) WMIF for MyHC (differentiated muscle) and GFP (ATRA-responsive cells) of control (F,G) and BMS493-treated embryos (H,I). BMS493 treatment before and after tamoxifen induction reveals a drastic decrease in GFP+ cells in the periocular region (I) compared with controls (G). Asterisks mark the location of the optic nerve (n = 3). (J) Coronal sections (dorsal, EOM origin) of E12.5 Tg:RARE-CreERT2;R26Tom;Scx-GFP embryos immunostained for GFP, Tom (ATRA-responsive cells) and MYOD/MYOG (muscle). Higher magnification views as insets. Arrowheads in J1 mark Tom-negative myogenic cells, and asterisks indicate sporadic labeling in myogenic cells. Arrowheads in J2 mark Tom+ Scx-GFP+ cells (n = 3). (K-L) WMIF for SMA (differentiated muscle) of MyodiCre (control, K,K’) and MyodiCre;R26RAR403 (mutant embryos, L,L’) (n = 3). EOMs in F-I and K-L’ were segmented from adjacent head structures and 3D-reconstructed in Imaris (Bitplane). a, anterior; ATRA, all-trans retinoic acid; ctrl, control; d, dorsal; E, embryonic day; EOM, extraocular muscle; l, lateral; m, medial; p, posterior; POM, periocular mesenchyme; Tam, tamoxifen; v, ventral; WMIF, whole-mount immunofluorescence. (TIF) [file pbio.3000902.s006.tif]

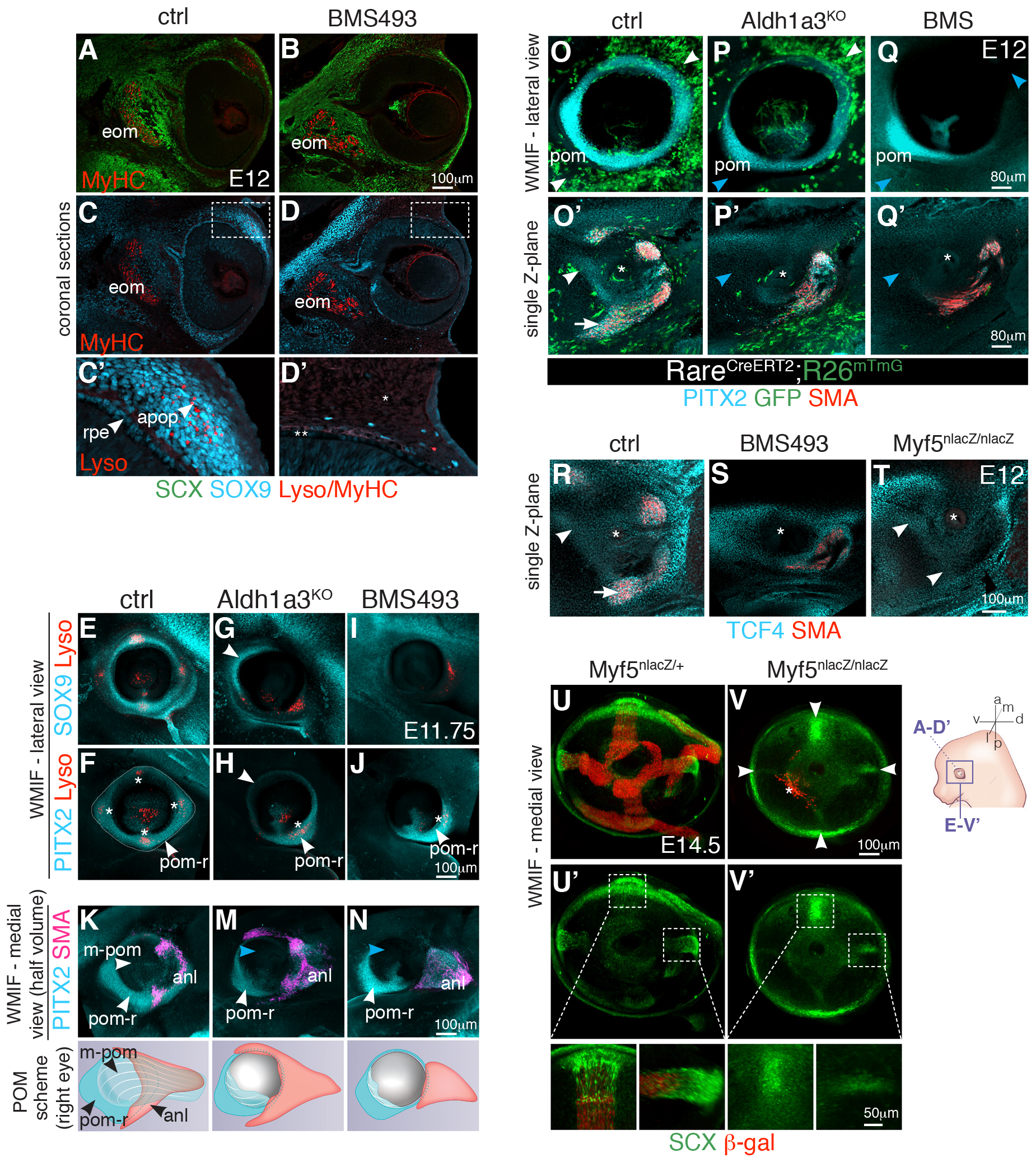

Supplement: S7 Fig — (A-D’) Immunostaining for the indicated markers on coronal E12 sections of control (A,C,C’) and BMS493-treated (B,D,D’) Tg:Scx-GFP embryos pre-incubated with LysoTracker Red (LysoR). (C’,D’) Higher-magnification views of the POM region. In BMS493-treated embryos, LysoR and SOX9 staining are absent in the POM (D’, asterisk) and SOX9 staining also missing in the RPE (D’, double asterisk). (E-N) WMIF for the indicated markers of E11.75 control (E,F,K), Aldh1a3KO (G,H,M) and BMS493-treated (I,J,N) embryos pre-incubated with LysoR (right eyes). In lateral views, neither Aldh1a3KO or BMS493-treated embryos (H,J) show a full PITX2+ POM-ring as the controls (F). Arrowheads in (G,H) mark remaining expression of SOX9/PITX2 in the POM of mutant or inhibitor treated embryos. Asterisks in (F,H,J) mark apoptosis spots in the POM. (K-N) Segmented medial views of periocular region of control, Aldh1a3KO or BMS493-treated embryos. Volumes were truncated in Z for clarity. Full view as schemes below. The PITX2+ POM-ring is continuous with the medial-POM in control embryos (K). In mutant and BMS493-treated embryos, residual PITX2 expression in POM is discontinous with the medial-POM (blue arrowheads) (M,N). (O-Q’) WMIF for SMA (differentiated muscle), GFP (ATRA-responsive cells) and PITX2 (muscle progenitors, POM) of Tg:RARE-CreERT2;R26mTmG control (O,O’), Aldh1a3KO (P,P’) and BMS493-treated (Q,Q’) embryos. Blue arrowheads in P and Q show reduction or loss of ATRA-responsive cells in the periocular region. (O’-Q’) Single Z-planes of the segmented volume. White arrowhead in O shows correct PITX2+ connective tissue pre-pattern in the prospective muscle areas and arrow marks PITX2 expression along the muscle masses. Blue arrowheads in P’ and Q’ show reduction or loss of ATRA-responsive cells and PITX2 pre-pattern in the medial periocular mesenchyme. (R-T) Single cell plane of WMIF of E12 control (R), BMS493-treated (S) and Myf5nlacZ/nlacZ embryos (T) (right eyes) for TCF4 (muscle connectiv [file pbio.3000902.s007.tif]

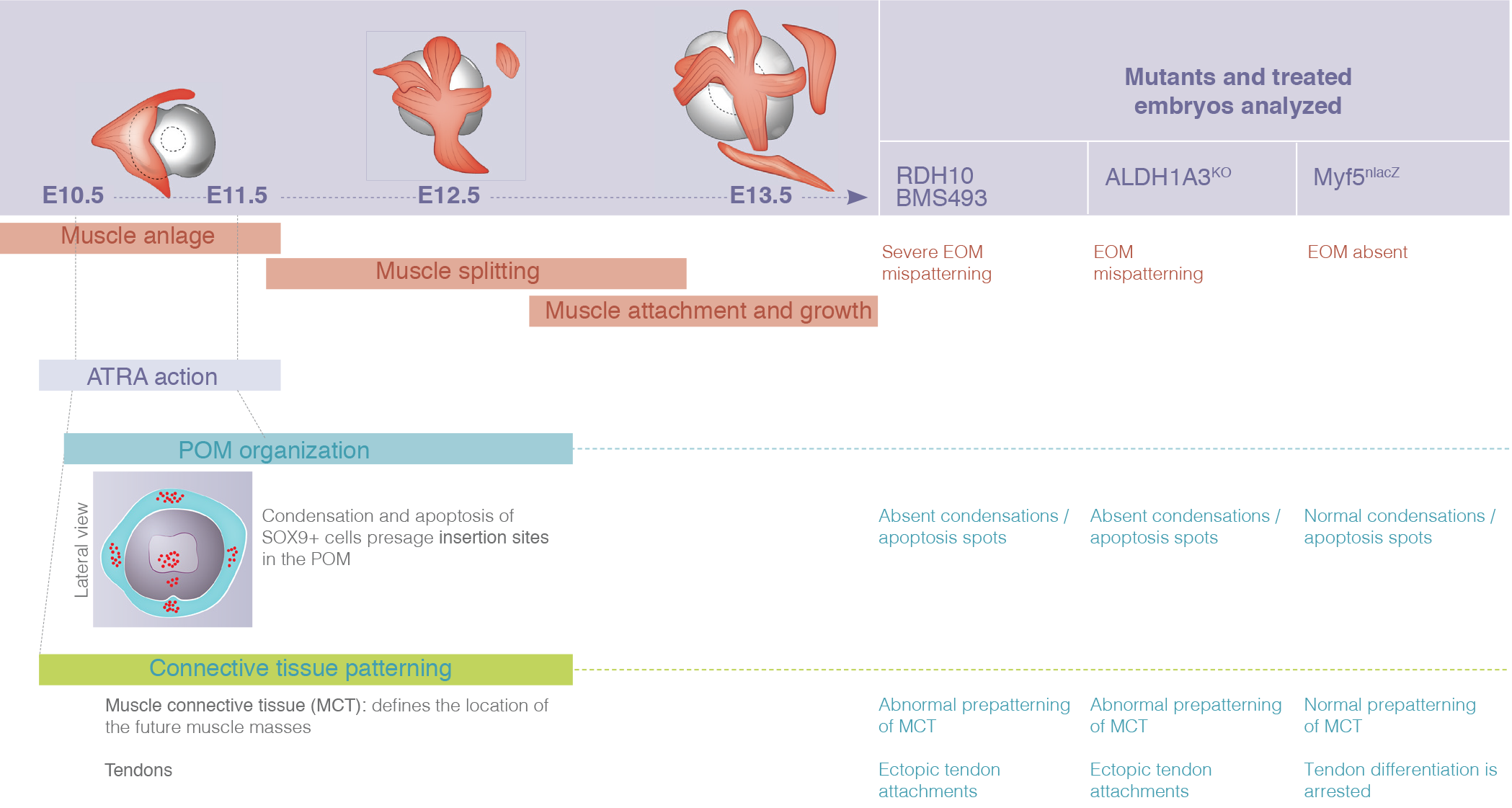

Supplement: S8 Fig — Summary of the most relevant genetic perturbations and drug treatments of this study. ATRA, all-trans retinoic acid; EOM, extraocular muscle; POM, periocular mesenchyme. (TIF) [file pbio.3000902.s008.tif]
