# Supplementary material for: Local retinoic acid signaling directs emergence of the extraocular muscle functional unit
Source: PLoS Biol. 2020 Nov 17;18(11):e3000902. doi: 10.1371/journal.pbio.3000902 (PMC7707851; doi:10.1371/journal.pbio.3000902)
Supplement: Attachment — Submitted filename: Response_reviewers.pdf [file pbio.3000902.s028.pdf]

**Revisions for manuscript # PBIOLOGY-D-20-00048R1**

**Text and figure changes in the revised manuscript:**

- **Modified text in revised manuscript in blue.**
- **Figure 1** has been reorganized according to Reviewers' comments for clarity. New data has been added as panels A1 and D1.
- **Figure 2** has been split into New Figure 2 and 3. New Figure 3 has been reorganized and new data added to panel K
- **Figure 3** has been combined with Suppl Figure3 and now presented as Suppl Figure 4
- **Figure 5** has been reorganized according to Reviewers' comments and new data has been added as panels F-F', G-I
- **Figure 6** has new high magnification views as insets in A'-G'
- **Suppl Figure 1** has new insets as A1-D1 and new data has been added as panels E-J
- Data in **Suppl Figure 2** is now presented in Suppl Figure 3D, and new data added as panels A-C in Suppl Figure 2 and panels A-C, E-H in Suppl Figure 3
- **Suppl Figure 4** has now become Suppl Figure 5
- **Suppl Figure 5** has now become Suppl Figure 6 and has new data as panels D, J, K-L
- **Suppl Figure 6** has now become Suppl Figure 7 and new data added as panels O-T
- A model summarizing the findings of this study has been added as **Suppl Figure 8**
- 4 additional **Videos** have been added

We thank the Reviewers and Editors for their enthusiasm regarding our work and for their comments which we believe have greatly improved the manuscript. Specific comments are addressed below in blue text. Much of the revision process, that required complex mouse crosses, was delayed due to the pandemic and mouse colony restrictions.

**REVISIONS**
**Reviewer 1:**
No questions to be addressed

**Reviewer 2:**
The manuscript describes well-constructed and executed series of experiments that address an important and fundamental question of how individual muscle units are constructed. Specifically, they address the formation of the extraocular muscles.
A key message from Figure 1 is that EOMs have connective tissue contributions from two distinct embryonic origins-neural crest laterally and cranial mesoderm medially. I found the presentation and description of the results quite hard to follow, to the point that the central message was lost/ less convincing to some extent. Specifically, L166 expression of markers is said to delimit a triangular area- I don't see this (I do see a clearer triangular area in panels E-H but this is not commented on). We thank the Reviewer for careful reading of our manuscript. While the findings remain unchanged, we have rewritten this section for clarity.

The Wnt1Cre lineage marker seems to label almost all cells in the sections shown. The region of muscle cells (labelled with Pax7/MyoD/Myogenin-shown in blue) appears negative -consistent with the muscle lineage being derived from cranial mesoderm but it is within the region that I assume we are supposed to also see mct (labelled as such in lower panel A) but this is not clear in this panel-a higher mag view could help.
We have now provided high magnification views as insets to unambiguously show that in ventral sections the Wnt1Cre lineage labels muscle connective tissue but it is excluded from myogenic progenitors (Figure 1A1).

The Scx-GFP that can be seen in this region in panel1B is not ideal as Scx is well established tendon marker and is expressed broadly in other regions of the section. It is problematic to use as an MCT marker in this context as the cells adjacent to the Muscle marker positive cells shown in panel A could be tendon cells rather than MCT. What could help here is some clear positive data showing co-labelling of Wnt1Cre R26Tom with an established mct marker.
We acknowledge the concerns of the Reviewer with regards to the use of Scx-GFP as tendon and connective tissue marker. We would like to clarify several points on this issue:

1. From an anatomical and histological point of view, all SCX+ cells cannot be exclusively tendon progenitors as they are also located in the middle of the muscle masses, and thus away from tendon insertion and tendon origin sites.
2. While the bHLH transcription factor Scleraxis (Scx) specifically labels differentiated tendons and its progenitors during embryogenesis, the early expression pattern of *Scx* in the developing limb mesenchyme is very broad, when no tendon-like tissue can be identified (Schweitzer et al. MOD 2003). Despite early detection of Scx progenitors, it is still not entirely clear whether the progenitor population induced at E10.5 are the source of cells for later tendons or whether these cells adopt connective tissue fates other than tendon (for example fascia or muscle connective tissues) (Huang, Dev Biol 2017). In the limb, a distinctive tendon pattern is not apparent until E12.5 when loosely organized tendon progenitors align between the differentiating cartilage and muscle tissues (Murchison et al., 2007). Moreover, there are several reports – that have probably received less attention in the field - describing *Scx* expression in muscle connective tissue fibroblasts in some epaxial muscles (Deries, Schweitzer & Duxson, Dev Dyn, 2010) and craniofacial mesenchyme (Cesario et al., Gene Expr Patterns 2016). In addition, in the developing chicken limb, *Scx* expression domains were found to be partially overlapping with those of the muscle connective tissue marker Osr1 (Stricker et al. Stem Cells and Dev, 2012). Finally, recent single cell transcriptomic data has shown that Scx-GFP is expressed in interstitial cells of adult limb muscles (Giordani et al. Mol Cell 2019).
3. Similarly, the "well-established MCT marker" TCF4, shows expression in cartilage (Witte et al. PNAS, 2010) and lower levels of expression in myogenic progenitors (Matthew 2011 et al. Dev 2011) during development, in addition to muscle connective tissue (MCT). Moreover, Tcf4 is also expressed in domains in which muscles do not form and which express Scx (Kardon et al., 2003). TCF4 expression becomes gradually restricted to MCT fibroblasts in late fetal stages and postnatal life (Matthew 2011 et al. Dev 2011).
4. To date, Tcf4 is the most commonly accepted marker for MCT fibroblasts, however Tcf4 exhibits a highly regionalized expression during development, which is mutually exclusive with other MCT markers such Osr1 in many places (Vallecillo Garcia, Nat Com 2017).

Together, we want to highlight the fact that connective tissue markers of specific cell types in the adult, show broad and partially overlapping expression patterns during development.

Therefore, as suggested by the Reviewer, we have performed a whole set of experiments to show that:
- MCT in the NCC-derived and mesoderm derived areas express TCF4 (Suppl Figure 1 E,F).
- TCF4 labels SCX+ cells distant from the tendon insertion and attachment points (Suppl Figure 1 G,H)
- SCX mRNA (RNAscope) is expressed in forming tendons but also abundantly in between myogenic cells distal from the tendon insertions (Suppl Figure 1 I,J).

On a more minor point, I think it would be helpful to label lower panels A' etc to help the reader navigate the figure. I think it would also help if for each data set using the NCC marker (Wnt1Cre) or mesoderm marker (Mesp1Cre) that comparable sections were shown with the reciprocal lineage marker ie. For panels A-B (ventral sections) that the staining obtained with Msp1Cre is shown and vice versa with panels E-H that the Wnt1Cre lineage marker is shown in the dorsal sections. This could help to clearly visualize the exclusion of cells of distinct embryonic origin to muscle origins and insertions.
We have reorganized the panel and now complementary sections as dorsal and ventral levels are shown for both Cre lines. To simplify the message, we have removed the panels showing single stainings and included high magnification insets of the MCT areas.

I found the schematic confusing/misleading as it shows two muscle domains when only one domain (posterior?) is visible in the IHC sections (Fig1A-D).
We have performed new cryosections on *Wnt1$^{Cre}$:R26$^{Tom}$:SCX-GFP* embryos to observe the muscle domains corresponding to superior and inferior rectus muscles in the same section as shown on the schematics. We like to highlight that given the complex anatomy of the EOMs, it is extremely difficult to get identical sections on different embryos and this is the major reason why most of our paper is based on analysis on 3D stainings.

I also found the key to the schematic hard to follow as the neural crest derived MCT is actually shown superimposed on the muscle and so appears as red dots rather than pink as shown in key

There seems to be a misunderstanding on the interpretation of color code of the schematic. Muscle is show as a red surface, and muscle connective tissue shows as pink dots in the NCC-derived area and violet dots in the mesoderm-derived areas as described in the key. We have slightly modified the schematics with bigger dots to make it visually simpler.

Another very minor point, dotted lines (167) are described as dashed (573) in figure legend. It is clearer for the reader if terminology is consistent.
This has been changed.

Fig2 and 3 provide detailed description of the formation of the EOM and how this can be disrupted in mouse mutants where the eye is abnormally formed. This study describes the normal formation and abnormal formation of the EOMs at greater resolution than has been described previously. The authors further use a novel RARE reporter combined with R26mTmG to track the lineage of RA-responsive cells. L329 onwards describe quantification of labelling and that minimal (co)labelling was detected in myogenic cells. It might be expected that the responsive cells are mct but this is not directly addressed.
We thank the Reviewer for raising this point. We have performed new experiments using the *Rare$^{CreERT2}$;R26$^{Tom}$;Scx-GFP* genetic combination to be able to address which connective tissue cell types (not exclusively mct) are responsive to ATRA. We found Tom+ cells in the *Scx-GFP+ cell* compartment (forming tendons and MCT as assessed per the validations of Figure 1 and Suppl Figure 1), SOX9+ cells or the periocular mesenchyme and CD31+ (endothelial) cells of the choroid and surrounding the optic nerve.

This is analyzed further using Scx-GFP as a marker of both tendon and connective tissue progenitors (Fig6). This is again problematic/a little unsatisfying as it is hard to be sure if the GFP cells being studied are tendon precursors or truly connective tissue progenitors. The text is careful to not over-state the results but would it be possible to use markers to distinguish mct in these regions more directly
These concerns have been extensively addressed in Figure 1 and Suppl Figure 1. Moreover, we have performed additional experiments including TCF4 (Suppl Figure 7 R-T) and Pitx2 (Suppl Figure O-Q') as connective tissue markers. We note that in the description of our results we mention periocular connective tissues of the periocular mesenchyme (including the POM ring providing tendon attachments, tendons, medial POM in between muscle masses, etc) which does include, but is not exclusively, muscle connective tissue.

Further comments:
Figure 1 could include DAPI staining to distinguish non-neural crest and non-mesoderm derived cells.
We have included the Hoechst channel on the overlay views.

Figure 1A bottom panel could show muscle staining to be consistent with the other bottom panels. We have reorganized the figure to accommodate comments from all Reviewers. The bottom panels with single stainings have been removed.

As stated above, the mct labelled in this panel is not obvious.
We have done a high magnification inset (Figure 1A1, D1)

Figure 1B. corneal epithelium is double labelled?
The corneal epithelium is not labelled with *Wnt1$^{Cre}$:R26$^{Tom}$:Scx-GFP*, but the corneal stroma is positive for Tom and SCX. However, as this has been described previously and is not the major point of the paper we have not included higher magnification images.

There is no structure called "corneal ectoderm". There is a surface ectoderm. However, by E13.5 the surface ectoderm does not exist. Prior to this stage, the surface ectoderm has detached from the lens and becomes the corneal epithelium. You can see the "corneal epithelium" faintly as the most anterior layer in the images shown. The area referred to as the "corneal ectoderm" are the neural crest cells, which differentiate into the keratocytes to lay down the corneal stroma.
The annotation of these structures has been changed on Figure 4. Results remain unchanged: The surface ectoderm is RALDH3 positive at E10.5 and the presumptive corneal epithelium is positive at E11.5. Corneal stroma is not annotated in the figure as it does not show RALDH3 labeling.

Labels are missing from some of the figures. E.g. Fig S4 SE? I also don't understand the CE label in this image?
Calling of the structure has been changed for Figure S4 panel A. Arrowheads do label the forming eyelid which has been called eyelid groove in the literature (Tao et al. Development 2005).

Why is ALDH1A3 staining visible in the corneal epithelium at E11.5 but not at E12.5 in the controls?
Indeed, expression of RALDH3 becomes downregulated in the corneal epithelium by E12.5 and becomes restricted to the eyelid groove (Matt et al. Development 2005 Figure 2 and Figure 3). The contrast of the image was adjusted so expression is properly visualized.

SE in fig 4 should have an arrow to the outermost layer of cells.
An arrowhead has been added for Figure 4A

The SE is stained on the nasal side but not the temporal, has a layer of cells been lost?
We performed new immunostaining on additional coronal sections of E10.5 embryos paying attention that histology was fully preserved. E10.5 image of Figure 4A has been replaced.

Fig. 6 shows type XII collagen staining, interestingly there is also a loss of expression in the corneal epithelium?
Collagen XII is not expressed in the corneal epithelium, it is expressed in the corneal stroma besides its expression in the periocular mesenchyme. Higher magnification images are now provided in Fig 6A-C.

MINOR TYPO
Line 80: space needed between NCC and independent
Line 85: "NCC cells" should be NCC
Line 244 "leds" should be leads?
Line 428 spelling "evolutionary"
L332 connective typo
We thank the Reviewer for their careful spotting of these typos. The misspelled words have been corrected throughout the text.

**Reviewer 3:**
This is a very interesting study that investigates developmental coordination of extraocular muscles (EOM), tendons, and their attachment sites in the sclera of the eye. Using genetic lineage analysis, the authors show that 1) the periocular connective tissue (muscle connective tissue and tendons) is of dual mesoderm/neural crest cell (NCC) origin and 2) the embryonic origin of the tendon insertion matches that of the muscle connective tissue. Using mouse genetics and inhibitor treatments, the authors go on to show that neural derivatives of the eye regulate morphogenesis of the EOM anlage and its scleral attachments by activating retinoic acid signaling in the NCC-derived periocular connective tissue. This study is exciting because it fills a gap in knowledge about the developmental mechanisms regulating tendon insertion into non-bone tissues. While I am enthusiastic about this work, I believe the study would benefit from some reworking (and possibly reorganization) of the results section to make it more accessible to the wide readership at PLOS Biology. In general, it was very hard to appreciate the significance of the results while keeping track of the many mouse mutants introduced.
We thank the Reviewer for pointing out the interest of our work. We have reorganized and simplified the results section to make it more accessible for a wider readership. We have removed the Pax6 data to Suppl Figure 4, together with Shh and Lhx mutants. Figure 1 has been simplified and Figure 2 split in New Figure 2 and 3.

Here are specific comments related to the results:
1. Since the embryonic lineage tracing data of the periocular connective tissue differs from what was previously published, it is important to show dorsal section of Wnt1-Cre and ventral sections of Mesp1-Cre at E13.5 in Figure 1 (the reciprocal sections in Supplemental Figure 1 are at E17.5).
We have added this data in Figure 1 as suggested by the Reviewer.

2. It is not made clear in the main text why chondrogenic factor Sox9 is being used. Based on the figure

legend, it seems as though it is being used as a marker for the dense fibrous connective tissue of the sclera. However, it could also be used to identify the tendon attachment sites. We agree with the Reviewer that the use of SOX9 as marker needed a better justification. This has been added in the text as on line 199 as:

*" In the developing limb and jaw, bone superstructures or ridges, generated by a unique set of progenitors that co-express SOX9 and Scleraxis (Scx), provide a stable anchoring point for muscles via tendons (Blitz et al., 2013; Blitz et al., 2009; Sugimoto et al., 2013; Roberts et al. Dev 2019). Although EOMs insert into a non-bone NCC-derived structure, early markers of pre-committed cartilage such as SOX9, are expressed in the POM (Thompson et al., 2010). To examine the time course of development of EOM insertions in greater detail, we immunostained Scx-GFP and Wnt1Cre;R26tdTomato;Scx-GFP coronal sections with PITX2 and SOX9 (Fig 3A-J'', Suppl Fig 3A-F')…."*

3. The sclera is not labelled in the figures (1, 2 , etc.) despite being listed in the legend.
We have corrected the figure legends.

4. Line 179: Either show the E11.5 data or delete statement about data not shown. We have removed this statement and tried to simplify the main message as suggested by the Reviewer.

5. Line 205: It is intriguing that apoptotic foci mark the future tendon attachment sites. What cells are undergoing apoptosis? It is difficult to see if these are Sox9/Pitx2 cells or another subpopulation of POM cells given the image magnification.
We have been intrigued by this result, yet found it very difficult to address this point given that most dying cells have a fragmented nucleus. Moreover, we could not perform Cleaved Caspase 3 antibody together with SOX9 immunostaining given that both antibodies working in our hands are raised in rabbit. However, we managed to capture Lyso+ SOX9+ cells and showed examples of those as insets in Figure2. Finally, we have now quantified the cell density in Lysotracker+ and adjacent Lysotracker- areas and found that in the former case cell density is twice higher. This observation strongly suggests that cell compaction may drive cell death and extrusion in this region of the POM.

6. Line 230: It is concluded that Sox9 and Scx have complementary patterns at the EOM insertion sites. It is important to know if these regions of overlap are composed of Sox9/Scx double positive cells that form the tendon-bone attachments in the mouse limb and jaw because the presence of Sox9/Scx double positive cells will suggest conservation of tendon attachment progenitor cells and strengthen the argument that some aspects of tendon-bone attachment are conserved during tendon-sclera attachment.
This is a very interesting point raised by the Reviewer. We have now provided higher magnification images clearly showing the presence of Scx-GFP+/SOX9+ (as well as SOX9+/Scx-GFP- cells) in the insertion sites between E11.5 and E13.5. The new data in Suppl Figure 3 shows that the remodeling of the POM is very dynamic, with SOX9+ condensates disappearing by E14.5 and SOX9 becoming restricted to the thin scleral layer. As such, our data suggest that transient SOX9 expression may represent a redeployment of the developmental module for tendon attachment, despite the fact there is no definitive cartilage in the mammalian sclera. This is an exciting observation that should be followed up in future genetic studies (as those performed in Blitz et al., 2013; Roberts et al. 2019).

7. Line 335: It is not made clear how the identity of the reporter-positive cells was determined to be connective tissue. Nor is it clear what is meant by "bulk cell preparations" in the main text or figure legend. Immunofluorescence followed by cell sorting? What marker was used to identify connective tissue cells?
We have clarified in the main text on line 334 as follows:
*"To assess if myogenic cells and adjacent POM cells respond to retinoic acid signaling, we microdissected the periocular region of RareCreERT2;R26mTmG embryos and subjected it to mild digestion in bulk. Cells were allowed to attach to culture dishes, immunostained, and scored for co-expression of GFP and myogenic markers (PAX7, MYOD, MYOG). Notably, the great majority of reporter positive-responsive cells were not myogenic (Fig 5B,C; Suppl Fig 6D)".*
Moreover, as discussed for the corresponding comment of Reviewer 2, we have performed new experiments using the *$Rare^{CreERT2}$;$R26^{Tom}$;Scx-GFP* genetic combination to be able to address which connective tissue cell types (not exclusively MCT) are responsive to ATRA. We found Tom+ cells in the

*Scx-GFP+* cell compartment (forming tendons and MCT), SOX9+ cells or the periocular mesenchyme and CD31+ (endothelial) cells of the choroid and surrounding the optic nerve.

8. In Figure 5, it is shown that NCC-specific loss of Rarb/Rarg leads to aberrant muscle morphogenesis (failure of splitting). Since the muscle connective tissue and tendons around the eye are of dual NCC-mesoderm origin, is it believed that RA signaling is specific to the NCC-derived connective tissue? Is the phenotype only restricted to regions in which the periocular connective tissue is NCC-derived? Is RA signaling important in the mesoderm-derived periocular connective tissue?
We thank the Reviewer for raising this relevant point. Disruption of retinoic acid signaling in Aldh1A3 KO, BMS treatments and NCC-specific loss of Rarb/Rarg results in abnormal muscle patterning and insertions in the POM, in a region that is purely NCC-derived. Given that the mechanism of EOM splitting was completely unexplored and concomitant to patterning of the respective tendon insertions in POM, we decided to focus on this aspect. The base of the anlagen, residing in mesoderm-derived CT, appears thickened in all three cases of retinoic acid deficiency and responsive to ATRA as per the *Rare$^{CreERT2}$;R26$^{Tom}$* readout (Suppl Figure 6J). However, we could not directly address this issue for several reasons. First, invalidation of retinoic acid signaling specifically in mesoderm-derived tissues was not feasible. *Mesp1$^{Cre}$:R26$^{RAR403}$* embryos are not viable (R.Kelly, IBDM, Marseille, personal communication) and *Mesp1$^{Cre}$:Rarb$^{fl/fl}$:Rarg$^{fl/fl}$* was not available in the lab. Instead, analysis of *Myod$^{iCre}$: R26$^{RAR403}$* (invalidation of ATRA signaling in myogenic progenitors) did not result in an over muscle patterning phenotype, as expected from the poor ATRA response of this cell type at this stage. Finally, as shown in Suppl Figure 5A'-A'', in the medial part of the POM RALDH2 is another source of ATRA, but inactivating its function with both *Wnt1$^{Cre}$* and *Mesp1$^{Cre}$* was out of the scope of this study.

9. Line 406: J' should be G'
Figure calling has been corrected

10. A summary figure at the end would be very helpful for the reader.
We thank the Reviewer for this suggestion, and have now added a scheme summarizing the major findings and mutants used in this study in Suppl Figure 8.

**Reviewer 4:**
In their manuscript, Comai and colleagues investigate the involvement of RA signaling in the genesis of extraocular muscle connections. They use mouse genetics and imaging to assess the developmental origins of the EOMs and their connective tissue. In contrast to other craniofacial muscle attachments, they find a dual neural crest cell and mesoderm origin to the connective tissue. They demonstrate that the EOMs form from a single anlage that subsequently splits. They show that attenuation of RA signaling either genetically or pharmacologically causes defects in the formation and insertion of the EOMs. They demonstrate that a short window of signaling (E10.75) is critical for the orientation of these muscle fibers and use a genetic reporter to characterize those cells that are RA-responsive. The schematics and supplemental movies that the authors use greatly assist in conveying complex anatomical details. The manuscript is well written and provides important new insights into an understudied aspect of biology. I have only a few concerns:
We thank the Reviewer for the encouraging comments.

1) The authors state that Sox9 and Scx expression patterns were segregated at E13.5 (lines 220-221). This is very difficult to appreciate from the images where it looks as if there is some co-expression. Given that Sox9;Scx co-expressing cells have been described in other systems, it would be worthwhile for the authors to determine if there is co-expression in the EOM connective tissue.
To address this point, we have now performed a time-course of expression of Scx-GFP and SOX9 in the POM between E11.5 and E14.5. We showed that between E11.5 and E13.5 there is co-expression of both genes at the tendon insertion sites (New Suppl Figure 3A-D), whereas SOX9 expression domain becomes progressively more restricted to the lateral-most POM and by E14.5 its expression becomes limited to the thin scleral layer. Future genetic experiments (e.g. targeted deletion of Sox9 in Scx-expressing eminence progenitor as in Blitz et al., 2013), would be required to assess the functional relevance of double positive cells for EOM attachment to the sclera.

2) For Figure 5, it would be more informative to show data from induction at E10.5 (rather than E 9.5), given that the authors show that E10.75 is a critical time window for RA signaling for EOM attachment.

From Suppl Fig. 5, it seems that the authors must have these data. Adding some sections from this time point could be informative as to the subpopulation of RA-responsive cells most important in patterning the EOM.

Indeed, this has been considered. Several reports in the literature indicating that the maximal recombination efficiency can be achieved between 12-24h upon Tamoxifen induction (Nakamura E. et al., Dev Dyn 2006; Nguyen M.T. et al., Dev Dyn 2009). Given that our BMS experiments showed that E10.75 is a critical time point for EOM patterning and attachment, we wanted to make sure that recombination had taken place before. We have now repeated the experiments where induction with Tamoxifen has been carried out at E10 (New Figure 5F-I).

3) Intro, line 86. McGurk et al. examined zebrafish not mice (the species is correctly stated in the discussion).

We apologize for - and corrected - this mistake in the introduction.
